# Supplementary material for: Optimizing success rate with Nonlinear Mapping Control in a high-performance raspberry Pi-based light source target tracking system
Source: PLoS One. 2025 Feb 25;20(2):e0319071. doi: 10.1371/journal.pone.0319071 (PMC11856289; doi:10.1371/journal.pone.0319071)
Supplement: Attachment — Submitted filename: Review Report.docx [file pone.0319071.s001.docx]

PONE-D-24-30172

**Optimizing Success Rate with Nonlinear Mapping Control in a High-Performance Raspberry Pi-based Light Source Target Tracking System**

The manuscript should be improved in the revised round according to the following comments.

Comment 1. The abstract is too long, it is recommended to precise the abstract. It is also suggested to include more recent advancements and provide novelty of the proposal.

Comment 2. In this proposal, the parameters Smax, Smin, Dmax, Dmin, and c have a critical role. It is recommended to highlight the impact of these parameters and selection criteria.

Comment 3. The limitation and discussion section should be expanded in the revised draft.

Comment 4. Add some latest studies from the recent literature on the application of the model. For example add Abbas et al. (2024), Abbas et al. (2024), and Abbas et al. (2024).

Comment 5. Provide some future recommendations related to the proposal.

Comment 6. It is recommended to thoroughly read the manuscript and avoid, typos, grammar, and spelling mistakes.

Comment 7. There are many abbreviations, it is recommended to add acronyms in the revised file.

Abbas, Z., Abbas, T., Nazir, H. Z., and Riaz, M. (2024). A novel adaptive CUSUM system for efficient process mean monitoring: An application in piston ring manufacturing process. *Alexandria Engineering Journal*, *106*, 87-100.

Abbas, Z., Nazir, H. Z., Abbasi, S. A., Riaz, M., and Xiang, D. (2024). Efficient and distribution-free charts for monitoring the process location for individual observations. *Journal of Statistical Computation and Simulation*, *94*(13), 2992-3014.

Abbas, Z., Nazir, H. Z., Xiang, D., and Shi, J. (2024). Nonparametric adaptive cumulative sum charting scheme for monitoring process location. *Quality and Reliability Engineering International*, [*https://doi.org/10.1002/qre.3522*](https://doi.org/10.1002/qre.3522).
